# Effects of Formalin-Inactivated Respiratory Syncytial Virus (FI-RSV) in the Perinatal Lamb Model of RSV

**DOI:** 10.1371/journal.pone.0081472

**Published:** 2013-12-06

**Authors:** Rachel J. Derscheid, Jack M. Gallup, Cory J. Knudson, Steven M. Varga, Drew D. Grosz, Albert van Geelen, Shannon J. Hostetter, Mark R. Ackermann

**Affiliations:** 1 Department of Veterinary Pathology, College of Veterinary Medicine, Iowa State University, Ames, Iowa, United States of America; 2 Interdisciplinary Graduate Program in Immunology, University of Iowa, Iowa City, Iowa, United States of America; 3 Department of Microbiology, University of Iowa, Iowa City, Iowa, United States of America; 4 Department of Pathology, University of Iowa, Iowa City, Iowa, United States of America; University ofTennessee Health Science Center, United States of America

## Abstract

Respiratory syncytial virus (RSV) is the most frequent cause of bronchiolitis in infants and children worldwide. There are currently no licensed vaccines or effective antivirals. The lack of a vaccine is partly due to increased caution following the aftermath of a failed clinical trial of a formalin-inactivated RSV vaccine (FI-RSV) conducted in the 1960’s that led to enhanced disease, necessitating hospitalization of 80% of vaccine recipients and resulting in two fatalities. Perinatal lamb lungs are similar in size, structure and physiology to those of human infants and are susceptible to human strains of RSV that induce similar lesions as those observed in infected human infants. We sought to determine if perinatal lambs immunized with FI-RSV would develop key features of vaccine-enhanced disease. This was tested in colostrum-deprived lambs immunized at 3–5 days of age with FI-RSV followed two weeks later by RSV infection. The FI-RSV-vaccinated lambs exhibited several key features of RSV vaccine-enhanced disease, including reduced RSV titers in bronchoalveolar lavage fluid and lung, and increased infiltration of peribronchiolar and perivascular lymphocytes compared to lambs either undergoing an acute RSV infection or naïve controls; all features of RSV vaccine-enhanced disease. These results represent a first step proof-of-principle demonstration that the lamb can develop altered responses to RSV following FI-RSV vaccination. The lamb model may be useful for future mechanistic studies as well as the assessment of RSV vaccines designed for infants.

## Introduction

Respiratory syncytial virus (RSV) is a common cause of acute lower respiratory infection and is the leading cause of infantile bronchiolitis worldwide [1], [2]. Premature infants, immunocompromised individuals and the elderly are at increased risk for severe infection and hospitalization. RSV vaccine development has been hampered since the 1960s when a clinical trial of a formalin-inactivated alum-precipitated RSV vaccine (FI-RSV) resulted in enhanced disease, two deaths, and hospitalization of 80% of the FI-RSV-vaccinated subjects [3]–[5]. Caution stemming from this incident has led to the development of animal models that mimic the FI-RSV vaccine-induced immunologic and pulmonary responses that follow RSV infection. Models have been developed in several species including BALB/c mice, Bonnet monkeys, macaques, Cotton rats, and cattle [6]–[13]. Much has been learned from these models with regard to the mechanistic basis underlying this enhanced host response, and the growing number of animal models that can mimic this condition suggests that the mechanism(s) may be universal across species.

Perinatal lambs have been increasingly used to study RSV pathogenesis, immunologic responses, and therapeutic strategies [14]. The lamb lung has several critical features conducive to modeling RSV infection in human infants, including similarities in branching patterns, numbers of Clara cells, cellular development, and innate immune responses. Perinatal lambs are also susceptible to human strains of RSV, develop similar lesions to human infants, and disease severity in lambs is increased in preterm compared to term and older lambs–as in humans [14]–[18]. Lambs can be born preterm and survive for RSV experimentation and therefore represent one of the few animal models that can be used for true comparisons to RSV disease in human infants born preterm. This is important because preterm birth is a leading risk factor for increased susceptibility to RSV-induced disease and hospitalization [1], [2]. Also, newborn lambs (preterm and term) can be deprived of colostrum thereby allowing studies lacking potential confounding effects of maternal immunoglobulin. It was our hypothesis that perinatal lambs vaccinated with FI-RSV would develop key features of the vaccine-enhanced disease previously observed in human infants. To determine the extent to which FI-RSV vaccination may alter clinical, pathologic, and immunologic responses in lambs, newborn lambs were vaccinated with a single dose of FI-RSV, challenged two weeks later with RSV for six days, then necropsied and assessed for clinical and pulmonary responses/parameters at six days following infection.

## Results

### Clinical Findings, Bronchoalveolar Lavage Fluid Viral Titers, Viral RNA Levels and Viral Antigen

**Figure 1:**
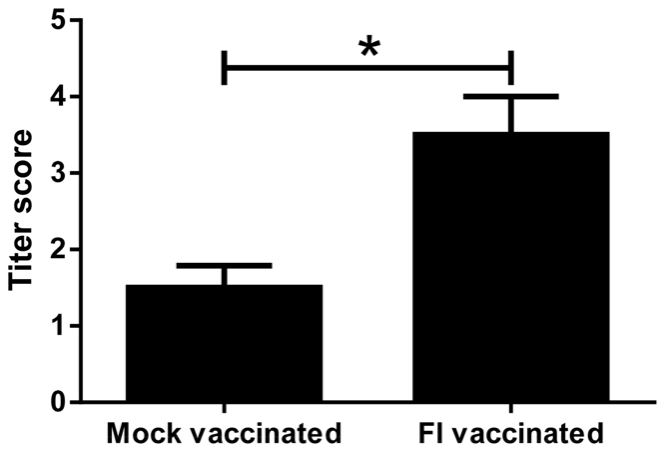
Serum neutralization titers of FI-RSV-vaccinated lambs (FI-vaccinated) (n = 4) were significantly increased compared to mock-vaccinated lambs (n = 4) (*P<0.05). Error bars = SEM.

There were no significant differences between RSV-infected lambs in terms of temperature, heart rate, body weight, respiratory rate, and cellular composition of the bronchoalveolar lavage fluid (BALF) according to scoring criteria we have used previously in newborn and preterm lambs [15], [16], [17], [18]. Sera from the FI-RSV vaccinated group had significantly higher neutralizing antibody titers than the control vaccine group, (p<0.05; Mann-Whitney test) (**Fig. 1**).

**Figure 2:**
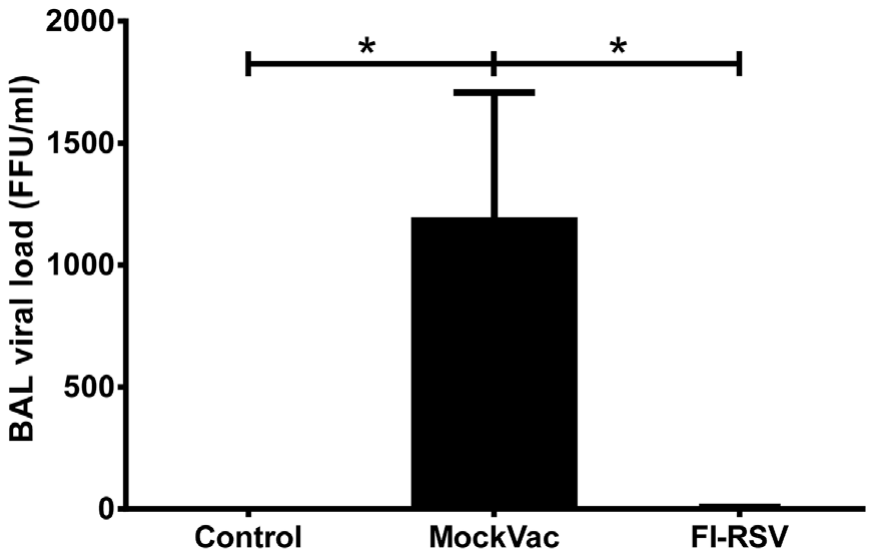
Bronchoalveolar lavage (BAL) titers of Control lambs (no vaccine and no RSV), mock-vaccinated (MockVac) lambs and FI-RSV-vaccinated lambs. FI-RSV-vaccinated lambs had significantly less RSV load compared to mock-vaccinated lambs. Control lambs lacked RSV titers. Control n = 4; MockVac n = 4; FI-RSV n = 4. Error bars = SEM, *P<0.05. FFU = focus-forming units per ml.

**Figure 3:**
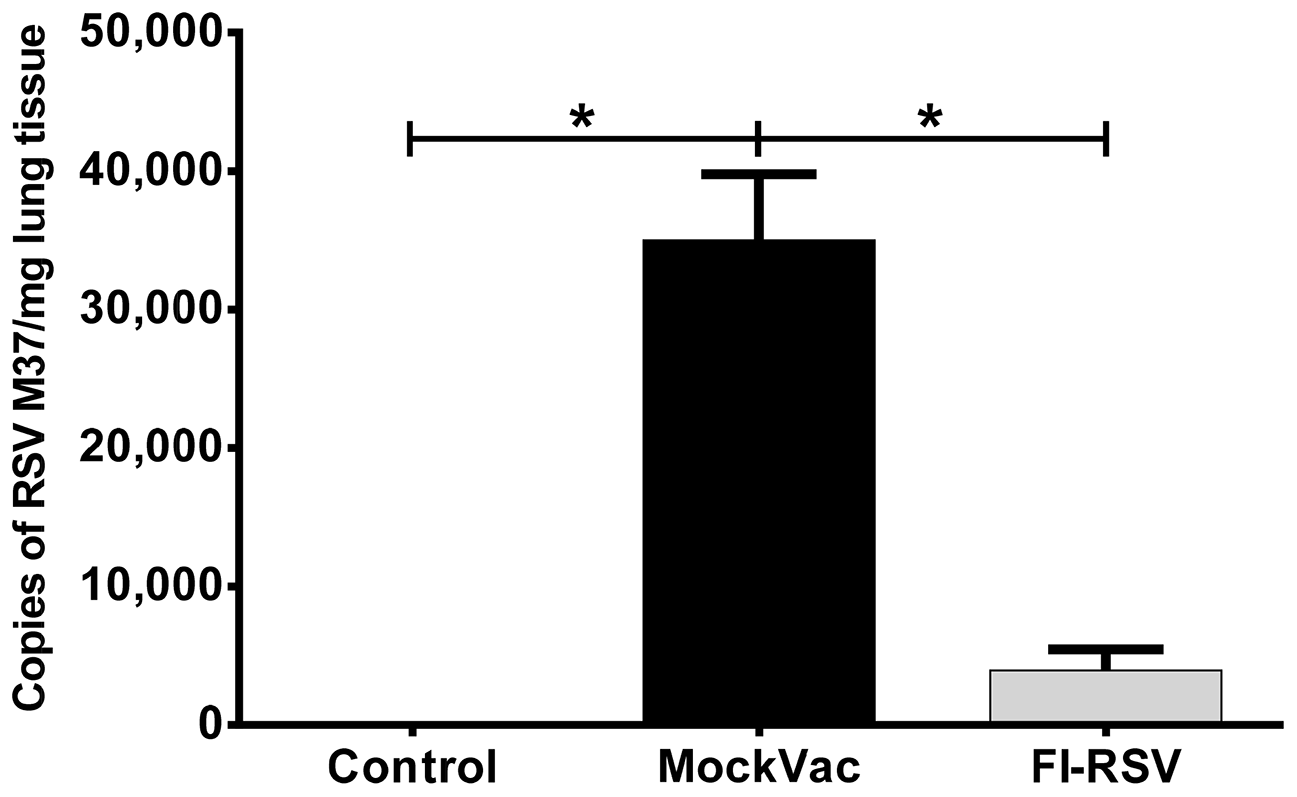
RSV RNA M37 levels in mock-vaccinated (MockVac), FI-RSV-vaccinated, and Control lambs as assessed by RT-qPCR. FI-RSV-vaccinated lambs had significantly less RSV M37 RNA compared to mock-vaccinated lambs. Control lambs lacked RSV RNA RT-qPCR signal. Control n = 4; MockVac n = 4; FI-RSV n = 4. Error bars = SEM, *P<0.05.

**Figure 4:**
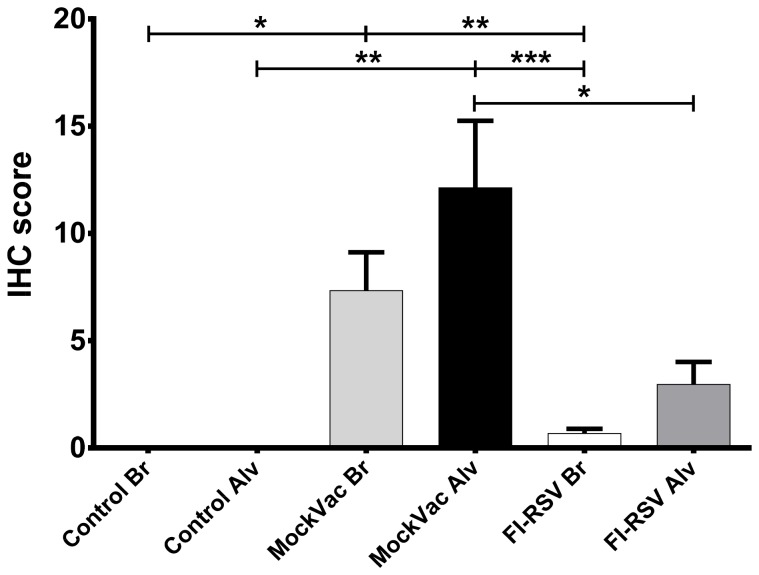
RSV antigen detected by immunohistochemistry (IHC). Control bronchioles and alveoli (Control Br and Control Alv) lack RSV antigen while bronchioles and alveoli of FI-RSV-vaccinated lambs had significantly reduced levels of RSV antigen compared to mock-vaccinated (MockVac) lambs. Control(s) n = 4; MockVac Br n = 4; MockVac Alv n = 4; FI-RSV Br n = 4; FI-RSV Alv n = 4. Error bars = SEM, *P<0.05, **P<0.01, ***P<0.001.

FI-RSV vaccinated mice exhibit reduced viral titers in the lung following RSV challenge [3], [11], [13]. Consistent with FI-RSV vaccination providing modest protection in mice, immunized lambs infected with RSV had significantly reduced (*P*<0.05) viral titers in the BALF (**Fig. 2**) compared to lambs that received mock vaccine. Viral titers in BALF from mock-vaccinated lambs reached 1190±517 FFU/ml, whereas BALF titers from FI-RSV lambs averaged less than 5 FFU/ml. Similarly, lambs vaccinated with FI-RSV and infected with RSV showed significantly reduced (*P*<0.05) RSV mRNA levels in lung tissue by RT-qPCR analysis (**Fig. 3**) compared to lambs that received a mock vaccine. Mock-vaccinated lambs averaged 35,005±4770 RSV copies/mg lung tissue whereas FI-RSV-vaccinated lambs averaged less than 3,950±1540 RSV copies/mg lung tissue. In both bronchi/bronchioles and alveoli, lambs vaccinated with FI-RSV and infected with RSV demonstrated significantly reduced (*P*<0.05) RSV antigen (**Fig. 4**) compared to lambs that received a mock vaccine following RSV infection. Bronchi and bronchioles had a score of 7.3±1.78 in mock-vaccinated lambs compared to RSV antigen levels of 0.67±0.22 in FI-RSV immunized lambs. Similarly, alveoli had a score of 12.1±3.12 for RSV antigen in mock-vaccinated lambs compared to a score of 3.0±1.05 in FI-RSV-vaccinated lambs. In summary, FI-RSV vaccination reduced RSV titers in BALF as well as RSV mRNA levels, and RSV antigen levels in the lung tissue.

### Histopathology

FI-RSV vaccinated children that died following a natural RSV infection exhibited bronchopneumonia, atelectasis, emphysema, dense lymphocytic and other mononuclear infiltrates around small (distal) bronchi, bronchioles, and involved alveoli [4]. In lambs, lesions present in those mock-vaccinated were identical to those described previously in lambs inoculated with RSV M37 strain and similar to those inoculated with RSV A2 and bovine RSV (bRSV) strains [7], [14], [15], [18]. Briefly, bronchioles contained infiltrates of neutrophils along with accumulation of cell debris and occasionally macrophages. Bronchiolar epithelium was eroded in some areas and there were individual degenerate cells that were slightly enlarged, partially detached from adjacent cells and had pyknotic nuclei; some nearby cells were necrotic. In the lamina propria and adventitia of bronchioles as well as multifocal small and medium-sized blood vessels were infiltrates of lymphocytes and plasma cells. There was moderate hypertrophy and hyperplasia of type II pneumocytes in alveoli, and alveolar lumens contained variable amounts of cell debris as well as occasional macrophages and moderate infiltrates of neutrophils. In lambs vaccinated with FI-RSV and inoculated with RSV, lesions differed significantly from those in mock-vaccinated lambs. In FI-RSV-vaccinated lambs, the bronchiolar epithelial lesions and infiltrates of neutrophils were present, but minimal with one to only several neutrophils in a few bronchioles.

**Figure 5:**
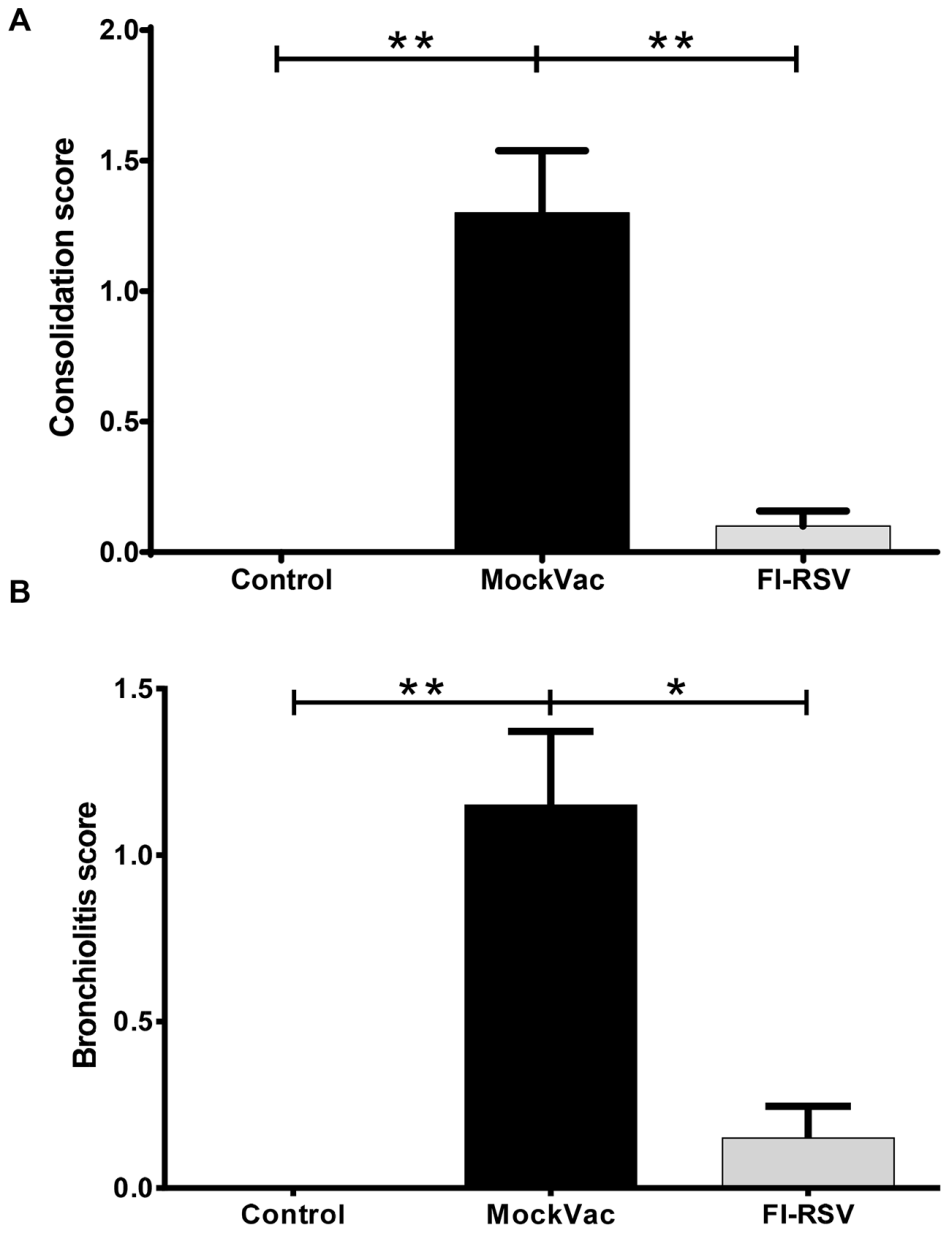
Histopathologic lesions of consolidation (A) and; bronchiolitis (B). Controls lacked lesions while mock-vaccinated (MockVac) lambs had significantly increased consolidation and bronchiolitis scores compared to lambs vaccinated with FI-RSV. Control n = 4; MockVac n = 4; FI-RSV n = 4. Error bars = SEM, *P<0.05, **P<0.01.

**Figure 6:**
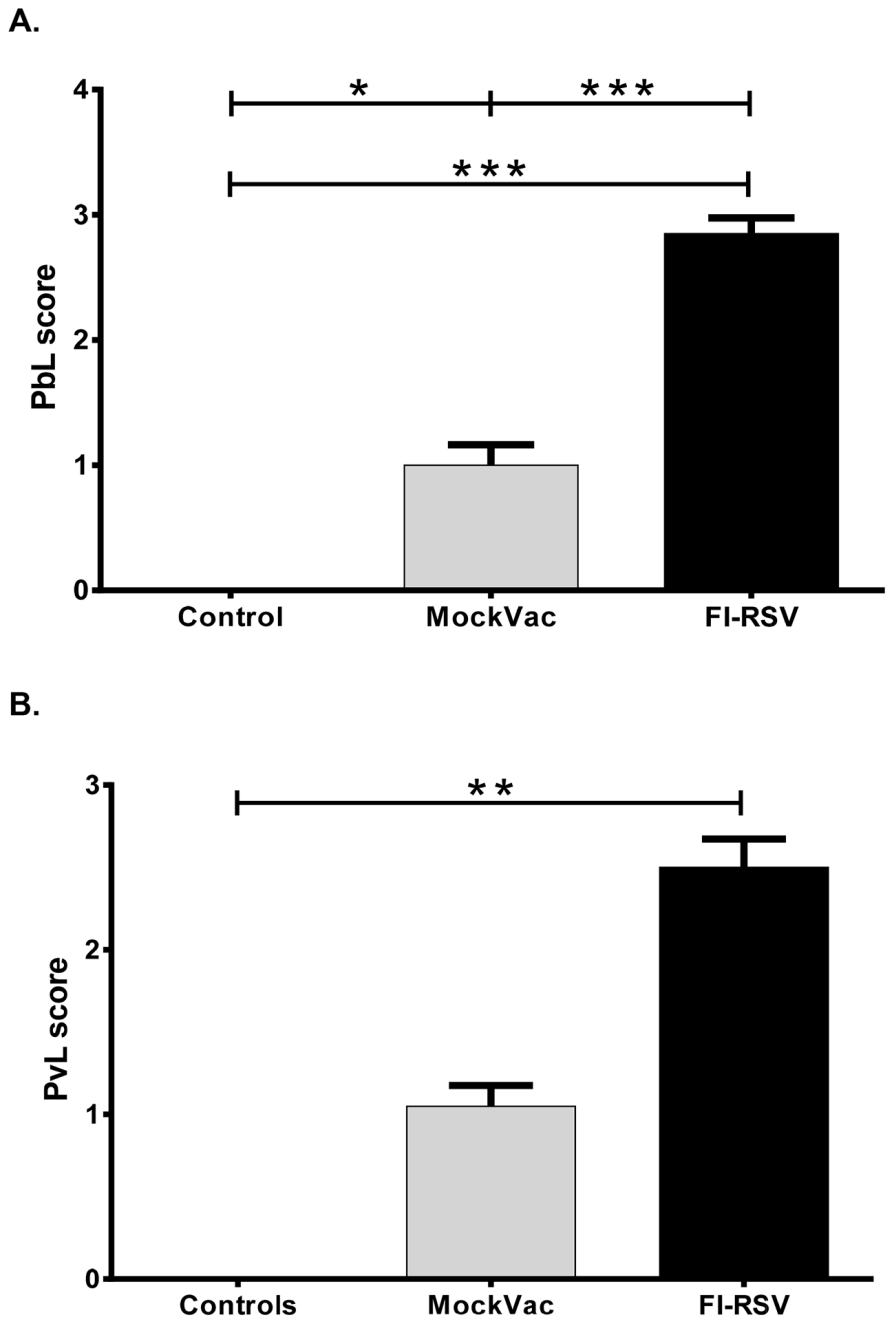
Extent of lymphocyte and plasma cell infiltrates into adventitia of bronchioles assessed by A. peribronchiolar lymphocytic (PbL) scores. FI-RSV-vaccinated lambs had significantly increased infiltrates compared to mock-vaccinated (MockVac) lambs. Controls lacked lesions. Extent of lymphocyte and plasma cell infiltrates into adventitia of blood vessels assessed by perivascular lymphocytic (PvL) scores (**B**). FI-RSV-vaccinated lambs had significantly increased infiltrates compared to control lambs. Mock-vaccinated lamb PvL infiltrates were not significantly different from control lamb levels. Controls lacked lesions. Control n = 4; MockVac n = 4; FI-RSV n = 4. Error bars = SEM, *P<0.05, ***P<0.001.

**Figure 7:**
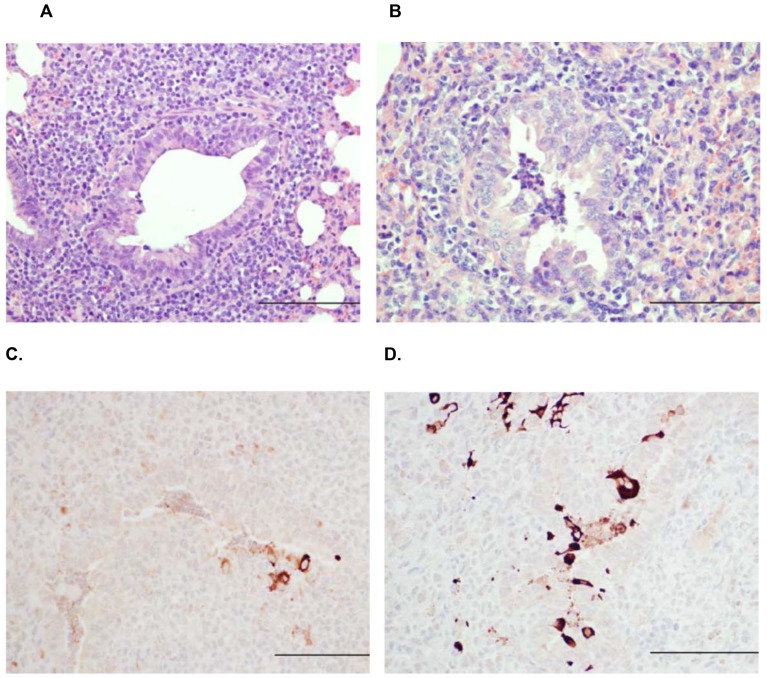
Lung of FI-RSV-vaccinated lamb with marked peribronchiolar lymphocytic infiltrates characterized by numerous, closely packed lymphocytes and plasma cells and a bronchiole that is slightly dilated but lacking intralumenal neutrophils (A) andLung of mock-vaccinated lamb with bronchiole containing neutrophils and relatively mild peribronchiolar lymphocytic infiltration characterized by a few lymphocytes and occasional plasma cells there are somewhat dispersed and not densely associated (B). Lung of FI-RSV-vaccinated lamb with marked peribronchiolar lymphocytic infiltrates and only a few cells containing RSV antigen (**C**). Lung of mock-vaccinated lamb with increased viral antigen distribution in multifocal cells and intensity characterized by more intense staining (**D**). Bar = 150 µm.

Lambs vaccinated with FI-RSV and infected with RSV had significantly reduced histopathologic scores (**Fig. 5**) compared to lambs receiving mock vaccine. Both bronchiolitis and consolidation scores were reduced significantly (**Fig. 5A, B**). In mock-vaccinated lambs, alveoli had a consolidation score of 1.3±0.23 while FI-RSV-vaccinated lambs gave a score of 0.1±0.06. Similarly, bronchiolitis scores were 1.15±0.22 in mock-vaccinated lambs as compared to 0.15±0.09 in FI-RSV-vaccinated lambs. Lambs vaccinated with FI-RSV and infected with RSV had significantly increased (*P*<0.05) lymphocyte and plasma cell infiltrates in airways and blood vessels (**Figs. 6A, B**) compared to lambs receiving mock vaccine. Lymphocyte and plasma cell infiltrates in the walls and adventitia of bronchioles, small bronchi, and adventitia of small and medium-sized blood vessels were more distinct and expanded due to increased cellularity. Lesion scores for bronchioles (PbL scores) were 2.85±0.13 in FI-RSV-vaccinated lambs and 1±0.16 in mock-vaccinated lambs. In blood vessels (PvL scores), lesion scores were 2.5±0.17 in FI-RSV-vaccinated lambs and 1.05±0.13 in mock-vaccinated lambs. Multifocal bronchioles had mild to moderate infiltrates of lymphocytes, plasma cells and occasional macrophages in the tunica adventitia. These infiltrates were also present in the lamina propria but to a lesser degree (**Fig. 7** **A, B**). Following RSV challenge, viral antigen was more prevalent in mock-vaccinated lambs than lambs vaccinated with FI-RSV (**Fig. 7** **C, D**).

## Discussion

FI-RSV-vaccinated lambs exhibited several key features of the FI-RSV-enhanced disease observed previously in human infants. These include the increased lymphocyte and plasma cell infiltrates around bronchi and bronchioles [4], [5] and pulmonary blood vessels the lesions in bronchiolar airways (bronchiolitis) and alveoli [4], [5], and the development of a moderate serum neutralization titer as well as significant reductions in BALF viral titers, viral mRNA, and viral antigen levels in the lung tissue. These findings demonstrate that the lamb develops altered responses to RSV following FI-RSV-vaccination. This study represents a simplified first attempt to assess the extent to which lambs may respond to FI-RSV immunization; additional studies will be needed to further optimize the model especially with regard to vaccine composition and timing of delivery, and mechanistically in regard to Th2/IgE responses.

The reason for the observed increase in lymphocytic and plasmacytic infiltration is not fully understood. Immune responses to FI-RSV have been shown to generate antibodies with low avidity to RSV and a reduced capacity to induce strong TLR4 responses [19], [20]. These effects, in addition to other unknown factors, likely contribute to a dysregulated immune response that triggers an exaggerated adaptive immune response with lymphocytes and plasma cells in large numbers and in a particular location of the lung (airway and blood vessel adventitia). In these locations, the cells produce a variety of chemokines, cytokines, and other immune/inflammatory mediators that affect bronchiolar epithelial cell activity and airway function, and the physical presence of these cells likely also affects airway bronchoconstriction and bronchodilation.

The lesions in bronchiolar airways and alveoli in mock-vaccinated lambs were similar to those caused by RSV A2 and bRSV in lambs and to those previously described in human infants and cattle [5], [7], [13], [21]. While vaccination with FI-RSV enhanced lymphocyte and plasma cell infiltration around bronchioles and blood vessels, bronchiolar airway and alveolar lesions were reduced in lambs vaccinated with FI-RSV. The reduced bronchiolar airway and alveolar lesions were associated with reduced RSV titers in bronchoalveolar lavage fluid and reduced RSV mRNA and RSV antigen. FI-RSV vaccination likely provided some degree of protection against bronchiolar airway and alveolar lesion development and helped to reduce viral replication. The serum neutralization titer was increased significantly compared to mock-vaccinated lambs and may have been enough to reduce RSV replication which, in turn, contributed to the observed reduction in bronchiolar and alveolar lesions. That is, the vaccine appeared to have a direct effect on viral load and an additional effect on an atypical immune response.

Eosinophils were not a prominent feature in any region of the lung. This lack of eosinophilic infiltration differs from findings in FI-RSV-vaccinated children as some of the vaccinated children exhibited eosinophilic infiltrates, as do some animal models affected by viral and non-viral antigens [10], [22]–[25]. On the other hand, the lack of eosinophils is similar to previous studies in FI-RSV-vaccinated cattle [7] and neutrophil infiltration was a prominent finding in Cotton Rats [12]. Sheep and cattle are both ruminants although the extent of similarities in their eosinophil function has not been fully determined. The role of eosinophils in the pathogenesis of FI-RSV lesions is not fully understood. It is important to point out that lesions were assessed in this study at 6 days post-RSV inoculation and, at later timepoints, lesions may be different. Also, the lambs were 3 weeks of age at the time of necropsy/evaluation, and even older lambs may exhibit different lesions as well. In addition, lambs in this study were inoculated with RSV two weeks after vaccination and natural infection in human infants occurred weeks to months after FI-RSV vaccination [4], [5]. Furthermore, animal models challenge at 3 to 5 or more weeks post FI-RSV vaccination [6]–[13]. The two-week timepoint in this study was used because the lambs were colostrum-deprived and there were concerns that the animals could be pre-disposed to secondary bacterial or other microbial infections. This did not occur in this study or in other studies we have on-going and therefore, future studies will be able to assess RSV challenge 3 or more weeks post-vaccination.

The lambs in this study lacked systemic signs of vaccine-enhanced disease. In this study, only one vaccine dose was administered, and only one timepoint was assessed after RSV infection. Also, this study used RSV A2 strain to make the FI-RSV vaccine while the lambs were challenged with a second strain, RSV Memphis strain 37, demonstrating that RSV A2-based vaccine preparation can yet result in FI-RSV-enhanced lesions. As indicated, additional studies will further optimize the model and can include: vaccine preparations that vary in protein content, use vaccine strains homologous to the RSV challenge strain, assessment of the effects of multiple vaccinations rather than a single vaccination, vaccination of lambs older than 2–3 days of age, determination of the kinetics of clinical, pathological and immunological responses at various timepoints following challenge, assessment of various delivery methods and doses of RSV, and the role of non-viral antigens. In addition, lambs can be born preterm and subsequently infected with RSV (17) resulting in disease severity and lesions that are increased compared to term lambs. This increased disease severity is similar to the increased susceptibility of preterm infants with severe RSV disease (4, 17). The effects of FI-RSV vaccination on preterm lambs may induce additional clinical, pathological and immunological responses.

This work demonstrates that FI-RSV vaccination of young lambs results in features consistent with enhanced disease in similarly vaccinated infants and other animal models. Because lamb lung and the lamb model of RSV infection have extensive similarities to human infants [14] the FI-RSV/lamb model may be useful in clarifying some of the mechanisms underlying the enhanced disease that occurs with RSV infection following FI-RSV vaccination and, potentially, other innovative vaccine formulations [28]. The model may be especially well-suited for studies of the potential effects of maternal antibody on FI-RSV vaccination since lambs can be deprived of colostrum (as in this study) thereby avoiding confounding issues related to the presence of maternal antibody [14]. The potential contribution of maternal antibody to RSV vaccination-related lesions is important in the development and testing of vaccines used in pregnant mothers and newborns. Also, unlike some other animal models, lambs can be born preterm, are susceptible to human strains of RSV, have developmental features and lung structure and cellular composition similar to human infants, and thus can be used for additional studies related to lung and immune system development.

## Materials and Methods

### Experimental Design

Colostrum-deprived lambs were acquired at 2–3 days of age and received vaccine or mock vaccine (1 ml) intramuscularly at 3–5 days of age. Three groups of lambs received the following: 1) no vaccine followed by nebulized media (no RSV), 2) mock vaccine followed by RSV inoculation (six ml of RSV M37 7.4×10^6^ FFU/ml in media with 20% sucrose via nebulizer over 23 minutes); and 3) FI-RSV followed by RSV inoculation. Lambs were euthanized and tissues were collected six days after RSV infection. This timepoint was selected because it results in peak lesions in this model [14], [15]. Clinical parameters (temperature, respiration rate, heart-rate and body weight) were logged daily, blood, bronchoalveolar lavage (BAL) fluid and lung tissue were collected six days after RSV infection, and RSV and lesion parameters were measured as described previously [15], [16]. All animal experiments were approved by the Institutional Animal Care and Use Committee (IACUC) of Iowa State University.

### FI-RSV Vaccine Preparation

A FI-RSV vaccine was made with RSV A2 strain as described previously [15]. Briefly, FI-RSV was prepared from RSV A2-infected Vero cells grown in OptiPROTM SFM media (Invitrogen, Carlsbad, CA). Virus-infected cells were removed by scraping followed by sonication for eight 1-second pulses, and subsequently centrifuged at 800×g for 10 minutes. Supernatant was inactivated with 10% buffered formalin at 1∶400 dilution for 72 hours at 37°C followed by ultracentrifugation at 50,000×g for 1 hour at 4°C. The pellet was resuspended in OptiPRO™ SFM at 1∶25 dilution with 4 mg/ml of Imject Alum adjuvant (Thermo Fisher Scientific, Waltham, MA). The solution was centrifuged at 1000×g for 30 minutes at 4°C and the pellet was resuspended at 1∶4 dilution in OptiPRO™ SFM (Gibco/Invitrogen). The FI-RSV preparation was sonicated in a water-bath sonicator for 15 seconds on ice and stored in amber glass vials at 4°C. An FI-mock vaccine was also prepared using the same protocol from a lysate of Vero cells mock-infected with PBS.

### RSV Serum Neutralization Assay

Sera of lambs were heat inactivated (40 minutes at 56°C) and added to DMEM with 5% heat inactivated normal sheep serum (NSS) to yield a total concentration of serum of 10% and the highest starting test serum concentration of 5%, or 1∶20. The sera were serially diluted in a two-fold series in DMEM 10% NSS. To each well, 50 FFU were added and the serum was incubated at 37% for 1 hr. The sera were placed in a 96 well plate with HEp-2 cells and incubated for 1 hr at 37°C, 5% CO2, after which 100 µl DMEM, 10% FBS were added and the plates were incubated for 5 days, then fixed with 3.7% formaldehyde in PBS, and counterstained with methylene blue. If the monolayer was intact, the serum dilution was scored as neutralizing, whereas lysed monolayers were scored as non-neutralizing. A numerical value was assigned to the neutralizing titer by multiplying the titer by 10, and titers lower than 1∶20 received a value of 1. A titer of 1∶20 was assigned a value of 2; 1∶40 was assigned a value of 4. Statistical significance was determined by the Mann-Whitney test.

### Administration of Virus to Lambs by Nebulization

PARI Sprint™ nebulizers were used to administer virus or control media to each lamb. Three 2-ml aliquots of virus-containing media or control media was administered to each animal over the course of 23 minutes resulting in the total inhalation of about 5 ml by each lamb. Preliminary studies showed that the addition of 20% sucrose was protective of the virus (manuscript submitted; Drew D. Grosz); therefore, the nebulate contained 20% sucrose.

### Tissue Collection and Viral Titers

After euthanasia the thorax was opened, lungs were removed, gross lesions were scored as described previously [17], [18] and photographed *in situ* and *ex vivo*. Tissue samples were collected from each lung lobe of all animals in the same manner. Briefly, multiple samples from each lobe were snap-frozen in liquid nitrogen for RNA isolation and subsequent reverse transcription quantitative polymerase chain reaction (RT-qPCR), two samples from each lobe were placed in tissue cassettes and put in 10% neutral-buffered formalin (NBF) for histological and immunohistochemical analysis, and two lung samples from each animal were placed into cryomolds with CRYO-OCT Compound (Tissue-Tek, Torrance, CA) then placed on dry-ice for transfer to −80°C storage for future cryo-sectioning. After removal, percentage parenchymal involvement was estimated for each lung lobe. Percentages were converted to a number using the following scale: 0% = 0, 1–9% = 1, 10–39% = 2, 40–69% = 3, 70–100% = 4. Group averages were calculated for the gross lesion score.

Bronchoalveolar lavage fluid (BALF) viral titers were measured for each lamb by flushing the excised right caudal lung lobe with 5 ml of cold modified Iscove’s media (42.5% Iscove’s modified Dulbecco’s medium, 7.5% glycerol, 1% heat-inactivated FBS, 49% DMEM, and 5 µg/ml kanamycin sulfate) after which 1 ml of the resulting BAL fluid was placed on ice for immediate use in our standard infectious focus assay. For this, 1 ml BALF sample collected from each lamb was applied to HEp-2 cells grown to 70% confluence in 12-well culture plates (Fisher Scientific, Hanover, IL) in DMEM media (Mediatech, Inc., Manassas, VA) supplemented to 10% with heat-inactivated fetal bovine serum (FBS) (Atlanta Biologicals, Atlanta, GA) and 50 µg/ml kanamycin sulfate (Invitrogen). Each sample was analyzed at full-strength and at four additional serial-dilutions of 1∶10, 1∶100, 1∶1,000 and 1∶10,000. 200 µl of each diluted BALF sample was assessed in duplicate. Overnight primary antibody (MAb to RSV Fusion Protein, Clone: RSV 3216 (B016), Meridian Bioscience, Cincinnati, OH) incubation was followed by washing and secondary antibody (Goat anti-Mouse Fab’ conjugated to AlexaFluor 488, Invitrogen) incubation for 30 minutes. Plates were rinsed and inspected for the presence of fluorescing foci of infection using the FITC/GFP filter on an inverted fluorescence microscope (Olympus CKX41, Center Valley, PA). Five or more fluorescing cells were counted as single focal events. Aliquots of BALF in PBS from accessory lobes were assessed for total nucleated cell count and cytology. Counts were performed using the ADVIA 120™ automated hematology analyzer (samples were diluted 1∶2 in isotonic saline prior to cell counting). Cytospin preparations of BALF were created using a Shandon Cytospin 3. Modified Wright’s was used to stain the slides and differential cell counts (based on a 300 cell differential) were performed by a board certified veterinary clinical pathologist blinded to the identity of all samples.

### Microscopic Lesion Evaluation and Scoring

A histologic score was determined by evaluating percent consolidation and converting the observed percentage ranges to a simple integer based on a consolidation scale used by our laboratory previously (35, 36): 0% consolidation = 0, 1–9% consolidation = 1, 10–39% consolidation = 2, 40–69% consolidation = 3, 70–100% consolidation = 4. Group averages were calculated for the alveolar consolidation score.

### Reverse Transcription Quantitative Polymerase Chain Reaction (RT-qPCR) Measurement of RSV M37 Nucleoprotein mRNA

Tissues from right and left cranial, right and left middle, and right and left caudal lung lobes (0.3–0.4 g of each) were homogenized in TRIzol (Invitrogen), pooled in equal portions to create a composite slurry for each animal, then adjusted to 0.091 g/ml with additional TRIzol. RNA isolation continued as per manufacturer’s instructions (Invitrogen), followed by DNase treatment (Ambion, TURBO DNase, Austin, TX) and 1∶10 dilution with a combination of RNaseOUT (Invitrogen) and ultrapure water (Millipore). NanoDrop (Thermo Fisher Scientific) was used to assess each sample for simple RNA purity and total RNA quantity (A260nm/A280nm all >1.95). Agilent Bioanalyzer 2100 analysis of total RNA prior to DNase treatment and dilution routinely showed RIN values of >8.0. RT-qPCR was carried out using One-Step Fast qRT-PCR Kit master mix (Quanta, BioScience, Gaithersburg, MD) in a GeneAmp 5700 Sequence Detection System (Applied Biosystems, Carlsbad, CA) with PREXCEL-Q calculations [26], [27]. Primers and probe for RSV M37 nucleoprotein were designed with ABI Primer Express 2.0 based on RSV accession number M74568. Forward primer: 5′-GCTCTTAGCAAAGTCAAGTTGAACGA; reverse primer: 5′-TGCTCCGTTGGATGGTGTATT; hydrolysis probe: 5′-6FAM-ACACTCAACAAAGATCAACTTCTGTCATCCAGC-TAMRA. Each 1∶10-diluted total RNA sample was further diluted so that each final RT-qPCR contained 0.784 ng total RNA/µl; which was determined optimal by PREXCEL-Q [19], [20]. Thermocycling conditions were 5 minutes at 50°C; 30 seconds at 95°C; and 45 cycles of 3 seconds at 95°C and 30 seconds at 60°C. Samples and standards were assessed in duplicate and each RT-qPCR quantification cycle (Cq) was converted to a relative quantity (rQ) based on a standard curve using the following equation: rQ = 10^((Cq−b)/m)^, where b and m are the y-intercept and slope, respectively, from a sample mixture-derived standard curve for RSV M37 nucleoprotein mRNA. The efficiency-corrected delta Cq (E_AMP_^ΔCq^) method was employed for RT-qPCR quantification analysis. Results were normalized to total tissue RNA loaded per reaction and identical for all reactions. No-RT control reactions proved negative for RSV M37. RT-qPCR was demonstrably free of inhibition based on PREXCEL-Q inhibitory dilution threshold analysis [19], [20].

### Detection and Quantification of RSV Antigen by Immunohistochemistry

Immunohistochemistry for detection, localization, and quantification of RSV antigen was performed on paraffin-embedded tissues using a method similar to what has been described previously [15]–[18], but with the following variations: instead of Pronase E antigen retrieval, heated buffer antigen retrieval was performed in TE-0.05% Tween 20, pH 9.0 in a pressure cooking device (Decloaking Chamber™ Plus, Biocare Medical, Concord, CA) using the factory default program (40 minutes with peak temperature (125°C) at 18 minutes and cooling to 80°C in another 22 minutes. Initial blocking for 15 minutes with 3% bovine serum albumin (BSA) (Fisher Scientific, Hanover, IL) in TBS-0.05% Tween 20, pH 7.4 (TBS-tw) was followed by additional blocking with 20% normal swine serum (NSS) (Gibco/Invitrogen) in TBS-tw for 15 minutes. Primary polyclonal goat anti-RSV antibody (EMD/Millipore/Chemicon, Billerica, MA) was applied (1.5 hours at 22°C) at a dilution of 1∶500 in TBS-tw containing 10% NSS and 3% BSA. After rinsing with TBS-tw, biotinylated rabbit anti-goat secondary antibody (Kirkegaard-Perry Labs, Gaithersburg, MD) diluted 1∶300 in TBS-tw containing 10% NSS and 3% BSA was applied (45 minutes), slides were rinsed, blocked with 3% peroxide in TBS-tw (25 minutes), followed by streptavidin-conjugated HRP (Invitrogen) diluted 1∶200 in TBS-tw (30 minutes). Color was developed using Nova Red (Vector, Burlingame, CA) for 90 seconds followed by water rinses, counterstaining (Harris’ hematoxylin, 2 minutes), counter staining with alkaline Scott’s water (1 minute), dehydration and cover-slipped with Permount medium (Sigma, St. Louis, MO). Slides (two lung samples) were scored: 20 unique 10X fields on each slide were assessed for antigen staining and immunoreactive cells were counted within bronchioles and alveoli. The number of cells immunoreactive for RSV per field was then assigned a score according to the following scale: 0 = 0, 1 = 1–10, 2 = 11–39, 3 = 40–99, 4 = >100.

### Statistical Analysis

Analyses were performed using GraphPad Prism 6 (GraphPad Software Inc, La Jolla, CA). All post-mortem data was assessed by one-way ANOVA followed by Tukey’s post-test. All clinical data were assessed by two-way ANOVA, and cumulative weight change was additionally assessed by one-way ANOVA followed by Tukey’s post-test. Serum neutralization titers were assessed by the Mann-Whitney test.
